# Shared genetics and causal relationship between sociability and the brain’s default mode network

**DOI:** 10.1017/S0033291725000832

**Published:** 2025-05-22

**Authors:** Giuseppe Fanelli, Jamie Robinson, Chiara Fabbri, Janita Bralten, Nina Roth Mota, Martina Arenella, Maroš Rovný, Emma Sprooten, Barbara Franke, Martien Kas, Till F. M. Andlauer, Alessandro Serretti

**Affiliations:** 1 Department of Biomedical and Neuromotor Sciences, University of Bologna, Bologna, Italy; 2 Department of Human Genetics, Donders Institute for Brain, Cognition and Behaviour, Radboud University Medical Center, Nijmegen, The Netherlands; 3 Global Computational Biology and Data Sciences, Boehringer Ingelheim Pharma GmbH & Co. KG, Biberach an der Riß, Germany; 4 Department of Forensic and Neurodevelopmental Science, Institute of Psychiatry, Psychology and Neuroscience, King’s College, London, UK; 5 MRC Cognition and Brain Sciences Unit, University of Cambridge, Cambridge, UK; 6 Department of Cognitive Neuroscience, Donders Institute for Brain, Cognition and Behavior, Radboud University Medical Center, Nijmegen, The Netherlands; 7 Groningen Institute for Evolutionary Life Sciences, University of Groningen, Groningen, The Netherlands; 8 Department of Neurology, Klinikum rechts der Isar, School of Medicine, Technical University of Munich, Munich, Germany; 9 Department of Medicine and Surgery, Kore University of Enna, Enna, Italy; 10 Oasi Research Institute-IRCCS, Troina, Italy

**Keywords:** brain activity, brain connectivity, expression quantitative trait loci (eQTL), imaging genetics, neuroimaging, resting-state functional magnetic resonance imaging (rs-fMRI), single-nucleus RNA sequencing (snRNA-seq), social withdrawal

## Abstract

**Background:**

The brain’s default mode network (DMN) plays a role in social cognition, with altered DMN function being associated with social impairments across various neuropsychiatric disorders. However, the genetic basis linking sociability with DMN function remains underexplored. This study aimed to elucidate the shared genetics and causal relationship between sociability and DMN-related resting-state functional MRI (rs-fMRI) traits.

**Methods:**

We conducted a comprehensive genomic analysis using large-scale genome-wide association study (GWAS) summary statistics for sociability and 31 activity and 64 connectivity DMN-related rs-fMRI traits (*N* = 34,691–342,461). We performed global and local genetic correlations analyses and bi-directional Mendelian randomization (MR) to assess shared and causal effects. We prioritized genes influencing both sociability and rs-fMRI traits by combining expression quantitative trait loci MR analyses, the CELLECT framework – integrating single-nucleus RNA sequencing (snRNA-seq) data with GWAS – and network propagation within a protein–protein interaction network.

**Results:**

Significant local genetic correlations were identified between sociability and two rs-fMRI traits, one representing spontaneous activity within the temporal cortex, the other representing connectivity between the cingulate and angular/temporal cortices. MR analyses suggested potential causal effects of sociability on 12 rs-fMRI traits. Seventeen genes were highly prioritized, with *LINGO1*, *ELAVL2*, and *CTNND1* emerging as top candidates. Among these, *DRD2* was also identified, serving as a robust internal validation of our approach.

**Conclusions:**

By combining genomic and transcriptomic data, our gene prioritization strategy may serve as a blueprint for future studies. Our findings can guide further research into the biological mechanisms underlying sociability and its role in the development, prognosis, and treatment of neuropsychiatric disorders.

## Introduction

Sociability, defined as the inclination to seek or engage in social interactions, is a complex trait that manifests as a continuum within the general population (Caldwell, 2012; Reeb-Sutherland, Levitt, & Fox, 2012). Various physical and mental health-related outcomes are influenced by sociability (Cacioppo et al., 2015). Particularly important for this study are the facts that social isolation is associated with mortality (Holt-Lunstad et al., 2015) and that social dysfunctions are relevant for neuropsychiatric conditions, including schizophrenia spectrum disorders, Alzheimer’s disease, autism spectrum disorder (ASD), and major depressive disorder (MDD) (Setien-Suero et al., 2022). Social dysfunctions constitute both a prodromal symptomatologic manifestation and a transdiagnostic negative prognostic factor (De De Donatis et al., 2022; Oliva et al., 2022).

Several brain structures have been proposed as neural substrates of social behavior (Porcelli et al., 2019), such as the precuneus/posterior cingulate cortex and the medial-prefrontal and temporal regions (Porcelli et al., 2019). These brain areas are central nodes of the default mode network (DMN), an integrated network implicated in various higher-order functions including social cognitive processes (Buckner, Andrews-Hanna, & Schacter, 2008; Raichle et al., 2001). The DMN’s components serve discrete yet integrated functions. The rostromedial prefrontal cortex and posterior cingulate cortex are pivotal for processing socio-cognitive information with relevance to the self (Leech & Sharp, 2014; Smith, Clithero, Boltuck, & Huettel, 2014). The medial temporal lobe, including the hippocampal formation, is essential for autobiographical memory processing, self-reflection, and the recollection of personal experiences (Andrews-Hanna, Smallwood, and Spreng, 2014; Spreng and Andrews-Hanna, 2015). The dorsomedial prefrontal cortex is integral to metacognitive functions, particularly those influenced by social context (Ferrari et al., 2016). Together with the temporoparietal junction, the dorsomedial prefrontal cortex enables mentalizing, i.e., the comprehension of mental states of other people (Andrews-Hanna et al., 2014; Spreng & Andrews-Hanna, 2015). The DMN’s role in self-referential cognition, theory of mind, the delineation of self from others, and autobiographical memory has been extensively documented (e.g., Andrews-Hanna et al., 2014; Spreng & Andrews-Hanna, 2015). Recent studies have particularly highlighted the DMN’s relevance to social behaviors in the context of major psychoses, underscoring its function as a central integrative hub for coordinating cognitive activities related to both self-directed thought and responses to external stimuli (Fox et al., 2017; Mulligan & Bicknell, 2023; Saris et al., 2020). Our previous work has elucidated DMN functional connectivity alterations as potential transdiagnostic markers for social dysfunction (Saris et al., 2022), thereby suggesting the utility for an augmented genetic understanding of the link between the DMN and sociability.

Functional MRI (fMRI) is a robust technique for mapping brain networks underlying social behaviour. While task-based fMRI measures the brain’s responses under controlled experimental conditions, resting-state fMRI (rs-fMRI) captures intrinsic, spontaneous fluctuations in neural activity that reflect the brain’s baseline functional architecture (Biswal, Yetkin, Haughton, & Hyde, 1995; Smitha et al., 2017). Therefore, rs-fMRI is particularly advantageous for studying the neural correlates of stable, trait-like behaviors such as sociability, as it does not depend on task compliance or external stimuli (Fox & Greicius, 2010; Raichle, 2006). Notably, the DMN is a prominent target of rs-fMRI research, as it is characterised through intrinsic connectivity patterns observed during rest (Sanz-Morales & Melero, 2024). Additionally, aspects of rs-fMRI-derived functional connectivity have been shown to be heritable, reliable, and comparable to task-evoked connectivity in detecting individual differences in brain organization (Elliott et al., 2019; Shah et al., 2016; Zhao et al., 2022).

Phenotypic correlations quantify the observed relationships between traits by capturing both genetic and environmental influences that contribute to their covariance. Nevertheless, phenotypic correlations do not disentangle the specific contributions of genetic factors. In contrast, genetic correlations estimate the extent to which two traits share a common genetic basis, although these estimates can still be influenced by factors such as population stratification, assortative mating, and misclassification bias (Bulik-Sullivan et al., 2015; Morris, Davies, Hemani, & Smith, 2020; van Rheenen, Peyrot, Schork, Lee, & Wray, 2019). This distinction is important for complex traits: phenotypic correlations are generally more driven by a combination of shared environmental exposures and genetic effects, while genetic correlations provide a more direct measure of shared heritability (Bulik-Sullivan et al., 2015). Empirical studies have shown that phenotypic correlations are reliable proxies for genetic correlations in traits with high heritability but are less concordant for behavioral or cognitive traits, such as sociability, where environmental influences play a larger role (Sodini, Kemper, Wray, & Trzaskowski, 2018). Thus, by focusing on genetic correlations, this study provides a robust framework for disentangling the shared genetic basis of sociability and DMN connectivity, which phenotypic correlations alone cannot achieve.

Sociability and functional brain networks are both influenced by genetic factors. The heritability of sociability-related behaviors, such as loneliness and social anxiety-related concerns, has been estimated at 48% (Boomsma et al., 2005; Stein, Jang, & Livesley, 2002). Specifically, loneliness and the fear of negative evaluation, a core feature of social anxiety, both showed a heritability of 48% in two large twin studies (Boomsma et al., 2005; Stein et al., 2002). Similarly, functional connectivity within the DMN has been found to be moderately heritable, with estimates around 42% (Elliott et al., 2019; Glahn et al., 2010). Genome-wide association studies (GWAS) have identified single-nucleotide polymorphisms (SNPs) at 18 independent genomic loci to be associated with sociability (Bralten et al., 2021) and 45 genetic regions as associated with brain functional signatures (Zhao et al., 2022). Zhao and colleagues reported the SNP-based heritability (*h^2^_SNP_*) for rs-fMRI node amplitude traits as 10.6–38.6%, and for functional connectivity traits as 3–60%. While previous research demonstrated a phenotypic association between sociability and the DMN, the underlying genetic correlations, shared associated genes, and potential causal relationships have not yet been explored.

In this study, we investigated the genetic relationships between sociability and DMN-related rs-fMRI traits by analyzing patterns of global and local genetic correlations and exploring potential causal relationships. Global genetic correlation measures the overall genetic similarity between two traits across the entire genome, providing an estimate of the proportion of variance shared due to common genetic variants; this assessment includes pleiotropic effects, where a single genetic locus affects multiple traits (van Rheenen et al., 2019). Local genetic correlation examines restricted genomic regions to identify where traits show significant genetic overlaps, deviating from the genome-wide average (van Rheenen et al., 2019). Additionally, we employed Mendelian randomization (MR) to assess potential causality between an exposure (e.g., sociability) and an outcome (e.g., DMN rs-fMRI traits). MR leverages genetic variants as instrumental variables to infer causal relationships, under the assumptions that these variants are associated with the exposure, not associated with confounders, and influence the outcome solely through the exposure (Sanderson et al., 2022).

An improved understanding of the genetic underpinnings linking the DMN and sociability may guide future research into the biological mechanisms underlying social behavior relevant to mental health and inform the development of therapeutic strategies for neuropsychiatric disorders. Thus, the aims of this study were, first, to characterize the shared genetic architecture and potential causal pathways between sociability and DMN-related rs-fMRI traits. Second, we aimed to prioritize genes associated with both types of traits via robust genomic and imaging analyses. Our approach leaves room for further replication and mechanistic validation in future translational work.

## Experimental procedures

### Input datasets

This study leveraged summary-level data from the largest available GWAS on sociability assessed from an aggregate score of UK Biobank self-report data (*N* = 342,461; Bralten et al., 2021), and GWASs on brain rs-fMRI traits from the UK Biobank (*N* = 34,691; Zhao et al., 2022) (Supplementary Table S1). Because the two GWASs originated from the same large-scale cohort, partial overlap of participants may occur. Further details on the cohort composition, recruitment strategies, and phenotypic assessment methodologies are available in the respective primary studies (Bralten et al., 2021; Zhao et al., 2022).

In the referenced GWAS by Bralten et al. (2021), sociability was operationalized as a composite score encompassing four self-reported items: (1) frequency of visits with friends or family, (2) number and type of social venues visited, (3) worrying after social embarrassment, and (4) feelings of loneliness. As for conceptual coherence, the first two items capture social interaction frequency and engagement in social environments, while the latter two address subjective aspects of sociability related to social anxiety and loneliness. The four items were combined into a single sociability score, where higher values indicate greater sociability. Items reflecting negative experiences (i.e., social embarrassment and loneliness) were reverse-coded to align with the overall construct (Bralten et al., 2021). Further details on the scoring methodology, and validation of this measure can be found in Bralten et al. (2021). Participants were excluded if they had confounding health conditions, such as extreme body mass index, neurological disorders, sensory impairments, or incomplete responses, to ensure data quality and reduce potential confounding factors (Bralten et al., 2021). For rs-fMRI traits, Zhao et al. (2022) leveraged pretrained spatial independent component analysis (ICA) loadings derived from previous work (Elliott et al., 2018) to map pre-processed rs-fMRI images from UK Biobank (see also https://www.fmrib.ox.ac.uk/ukbiobank/). A total of 76 ICA-derived node amplitude traits were generated, each corresponding to specific regions of intrinsic neural activity. These nodes were spatially localized to anatomical regions using the Automated Anatomical Labelling Atlas 3 (Rolls et al., 2020) and then labelled to major functional networks using established network definitions from Finn et al. (2015) and Yeo et al. (2011). Functional connectivity traits, referred to as ‘edges’, were calculated as temporal correlations of BOLD signal fluctuations between pairs of ICA-defined nodes. The full set of connectivity traits included 1,695 pairwise measures of coactivity between nodes, which were further summarized into six global network connectivity dimensions, capturing broad patterns of functional interactions across the brain (Zhao et al., 2022). Rs-fMRI was chosen for its capacity to capture intrinsic neural activity and functional connectivity independently of task performance or external cognitive demands. By measuring spontaneous low-frequency BOLD fluctuations in neural activity, rs-fMRI provides stable, trait-like representations of connectivity patterns that are ideally suited for investigating broad, transdiagnostic behavioral phenotypes, such as sociability. This approach minimizes variability associated with task-specific compliance or engagement, making it an effective tool for large-scale cohort studies, such as the UK Biobank (Elliott et al., 2019; Fox & Greicius, 2010). To focus on the DMN, we selected traits from Zhao et al. (2022)‘s GWAS dataset based on the following criteria: traits mapping to the DMN, demonstrating a significant *h^2^_SNP_* ≥ 0.1, and with genome-wide significant loci (available for download at https://zenodo.org/records/5775047). Connectivity metrics for DMN traits were confirmed to include at least one node aligning with a DMN template, consistent with standard ICA-derived parcellations used in rs-fMRI studies (Zhao et al., 2022). Importantly, our study does not assume exclusivity of DMN involvement in sociability but rather adopts an *a priori* hypothesis-driven approach, while recognizing that genetic influences on sociability likely extend beyond the DMN to other functional networks.

All utilized GWAS summary statistics included only individuals of European ancestry and used the reference genome build GRCh37/hg19. For the sociability GWAS, regression coefficients representing the effect sizes were adjusted for covariates such as sex, age, and principal components during linear regression analyses (Bralten et al., 2021). For the rs-fMRI traits, regression models were adjusted for a comprehensive set of covariates, including age (at imaging), age-squared, sex, age–sex interaction, age-squared–sex interaction, imaging site, head location, head motion, head size, long-term drifts, and the top 40 genetic principal components (Zhao et al., 2022). Our analyses used these effect sizes as reported in the original publications.

Ethical approval and participant informed consent for the UK Biobank studies were obtained as per the original GWAS publications (Bralten et al., 2021; Zhao et al., 2022).

### Genetic overlap and causal relationships between sociability and rs-fMRI traits

**Figure 1.**
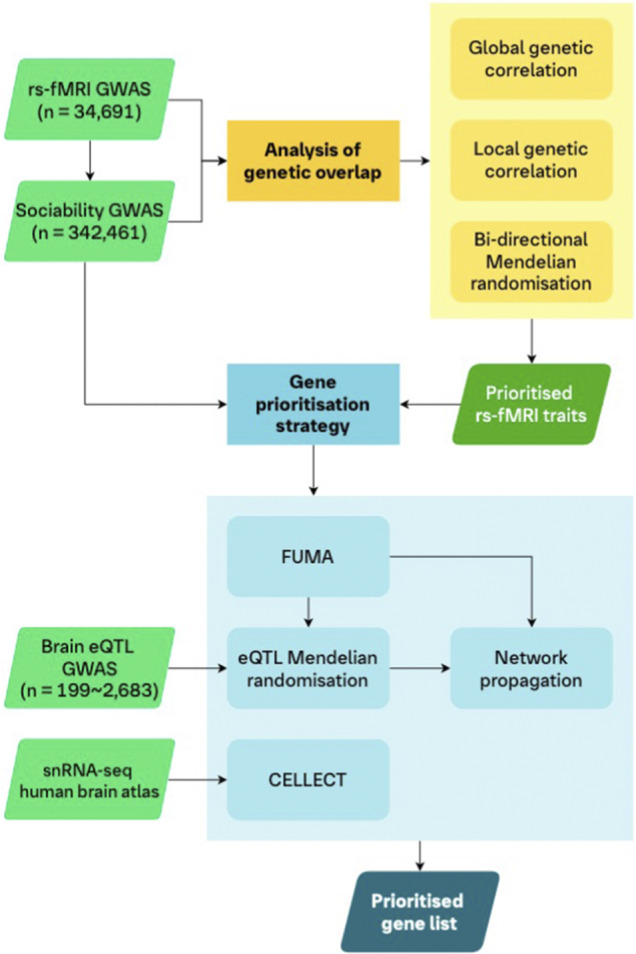
Analysis workflow. A schematic of the workflow of our analyses. We utilized genetic correlations and bi-directional MR to assess the genetic overlap between rs-fMRI traits and sociability to prioritize selected rs-fMRI traits for the downstream gene prioritization strategy. First, the GWAS of the prioritized rs-fMRI traits and sociability were analyzed using FUMA to map associated genetic regions to genes. We then leveraged eQTLs of gene expression in five brain tissues in an MR framework to provide further putative causal evidence for the mapped genes. Genes from these mapping steps were included in a TieDIE network propagation analysis using the underlying STRING protein–protein interaction network. Separately, we also integrated a human brain transcriptomics atlas (snRNA-seq data) in a CELLECT framework with the rs-fMRI and sociability GWAS. This step allowed us to identify genes whose increased expression are specific to cell types, in specific brain regions, for our traits of interest. Our final list of prioritized genes consisted of those genes which were identified by FUMA and showed at least nominal evidence in both the eQTL MR and CELLECT analyses, for both sociability and at least one rs-fMRI trait. Finally, we used the TieDIE network propagation scores to rank the list of prioritized genes.

We explored the genetic overlap and potential causal relationships between sociability and rs-fMRI traits related to the DMN using global and local genetic correlation analyses, as well as bi-directional MR analyses (see Analysis workflow, Figure 1). All rs-fMRI traits showing significant associations with sociability after correction for multiple testing in either correlation analyses or bi-directional MR analyses were selected for gene prioritization.

#### Global and local genetic correlation analyses

Bivariate Linkage Disequilibrium Score Regression (LDSC) analyses (Bulik-Sullivan et al., 2015; https://github.com/bulik/ldsc) were conducted to estimate the global genetic correlation (*r_g_*) between sociability and rs-fMRI traits using default LDSC parameters. LDSC is computationally robust even when sample overlap between GWASs occurs (Bulik-Sullivan et al., 2015). Only the sociability and rs-fMRI trait pairs showing at least nominally significant (*p* < 0.05) global, bivariate genetic correlations were further explored at the local genomic level.

Pairwise local genetic correlation analyses were conducted using LAVA (Local Analysis of [co]Variant Association), using standard parameters (Werme, van der Sluis, Posthuma, & de Leeuw, 2022; https://github.com/josefin-werme/LAVA). In contrast to global correlation analyses, LAVA enables the identification of genomic regions that may be implicated in shared genetic etiology and thus provides a fine-grained perspective on the genetic sharing between complex traits. Its analytical framework allows the investigation of genetic associations between traits under varying genetic causality scenarios without assuming a specific distribution of SNP effects (Werme et al., 2022). In summary, we generated 2,495 semi-independent genomic regions of approximately 1 mega base-pair length, and only genomic regions with significant local *h^2^_SNP_* (univariate *p* < 1 × 10^−04^) for both phenotypes were analyzed (Werme et al., 2022). To account for overlapping samples among input GWASs, we supplied LAVA with intercepts derived from bivariate cross-trait LDSC analyses (Bulik-Sullivan et al., 2015); these intercepts served as estimates of the sampling correlation between the datasets.

Because the rs-fMRI traits were not fully independent of each other, we applied the Benjamini-Hochberg false discovery rate (FDR) correction for multiple testing, considering a maximum acceptable FDR of *q* = 0.05 (Benjamini & Hochberg, 1995). Applying Bonferroni correction in this context would be overly conservative, increasing the likelihood of false negatives (Type II error). FDR correction accounts for dependencies among traits (e.g., connectivity edges inherently correlate with the activity of their corresponding nodes) and is widely adopted for genetic correlation analyses (e.g., Hindley et al., 2022; Hu et al., 2024; Qi et al., 2024).

#### Bi-directional Mendelian randomization

We assessed potential causal relationships between sociability and DMN-related rs-fMRI traits using bi-directional MR. This method utilized genetic variants as instrumental variables under three core assumptions: relevance, independence, and exclusion restriction (Sanderson et al., 2022). Here, we parameterized two SNP instrument selection criteria following a paradigm employed previously to investigate a link between imaging-derived phenotypes and Alzheimer’s disease (Knutson, Deng, & Pan, 2020): (1) a stringent analysis using only robust and independent instruments (*p* < 5 × 10^−08^, clumping threshold *r^2^* < 0.001), and (2) a lenient analysis using weakly-correlated instruments (*p* < 5 × 10^−05^, clumping threshold *r^2^* < 0.1). The rationale for this second approach was to increase the proportion of phenotype variance explained by the genetic variants and thus to increase statistical power (Knutson et al., 2020). Because these analyses were considered a complementary approach to genetic correlation analyses, sociability and all 95 DMN-related rs-fMRI traits were included in the MR assessments. Multiple testing correction was applied using Bonferroni’s method based on the number of tested traits (significance threshold *α* = 0.05/96 = 5.21 × 10^−04^). MR relies on independent genetic instruments, and Bonferroni correction aligns with the stringent standards required to minimize the risk for false-positive causal claims in MR studies (Sun, Wang, & Kan, 2024; Wu, Huang, Hu, & Shao, 2020; Zhang et al., 2024).

#### Mendelian randomization statistical methods

We used the Wald ratio for exposures with a single SNP instrument (overwhelmingly used in the eQTL MR, see below) or the inverse variance-weighted (IVW) method for exposures with multiple instruments (overwhelmingly used in the bi-directional MR, see above). However, we modified these methods to increase their utility for our use cases. First, we used the two-term Taylor series expansion of the Wald ratio to account for the error in both the instrument-exposure and instrument-outcome relationships. Second, we used an extended IVW method which allows for correlated instruments in the lenient bidirectional MR analyses (Burgess, Dudbridge, & Thompson, 2016). If a genetic exposure variant did not match the rsID of an outcome SNP, we searched for a proxy variant with a threshold of *r^2^* > 0.8 (applies to all analyses).

MR relies on three core assumptions: (1) the genetic instruments are strongly associated with the exposure (relevance), (2) the instruments are not associated with any confounders of the exposure-outcome relationship (independence), and (3) the instruments affect the outcome solely through the exposure and not via any alternative pathways (exclusion restriction) (Sanderson et al., 2022). To account for weak instrument bias, we specified that all instruments require an F-statistic of at least 10 (Bowden et al., 2016). As a sensitivity analysis for exposures with multiple instruments, we used an MR-Egger regression framework to assess the likelihood of the presence of horizontal pleiotropy. In the weakly correlated instruments analysis, we used the extended method described by Burgess, Dudbridge, and Thompson (2016). When analyzing single genetic instruments, we examined evidence for reverse causation using Steiger filtering to assess whether the genetic variant explained more of the variance in the outcome than in the exposure (Hemani, Tilling, & Davey Smith, 2017). Analyses were conducted using a custom implementation based on the *TwoSampleMR* R package (Hemani et al., 2018) in R version 4.1.0 (Team, 2021). This study was not formally pre-registered; however, our analytical plan, including the choice of instruments, thresholds, and sensitivity tests, was established prior to data analysis and is explicitly detailed here for transparency. We adhered to the Strengthening the Reporting of Observational Studies in Epidemiology (STROBE) guidelines for MR (MR-STROBE) to ensure comprehensive and transparent reporting of MR methodology and interpretation of findings (Skrivankova et al., 2021).

### Identification of genes from GWAS loci

To identify genes associated with both sociability and the selected rs-fMRI traits, we conducted several complementary analyses providing causal, expression, and biological evidence (Figure 1). As a first step, we generated a list of potentially associated genes from the summary statistics of each GWAS using FUMA (Functional Mapping and Annotation of GWASs; Watanabe, Taskesen, van Bochoven, & Posthuma, 2017), followed by providing causal evidence for the association of these genes using expression quantitative trait loci (eQTL)-based MR.

#### FUMA-based gene mapping

We ran FUMA using default parameters for both sociability and the rs-fMRI traits prioritized in the genetic correlation and MR analyses to identify genes linked to the respective genome-wide significant loci. Only brain-derived tissue types were used for eQTL gene mapping. For prioritization, genes had to meet any of the following criteria from the FUMA analyses: (a) FDR-corrected MAGMA *p* < 0.05; (b) FDR-corrected eQTL mapping *p* < 0.05; (c) implicated in 3D chromatin mapping.

#### Mendelian randomization of gene expression

Using eQTL-based MR, the FUMA-prioritized genes were tested for evidence of a causal relationship with either sociability or the prioritized rs-fMRI traits. To this end, we estimated the putative causal effect of genetically proxied gene expression on genetic proxies of sociability and rs-fMRI traits. We utilized eQTLs from the MetaBrain resource, a meta-analysis of brain-derived eQTLs across five different tissues (basal ganglia, cerebellum, cortex, hippocampus, and spinal cord) (de Klein et al., 2023).

We constructed *cis*-acting genetic instruments (as *trans*-acting eQTLs are more liable to pleiotropy) with a recursive *p*-value selection paradigm. We first searched for an eQTL for a gene at *p* < 5 × 10^−08^ and, if no eQTL was found, we reduced this threshold to *p* < 5 × 10^−07^ and, finally, to *p* < 5 × 10^−06^. If a gene was part of an eQTL with *p* < 5 × 10^−08^, we did not include genetic variants with eQTL *p*-values in the range 5 × 10^−08^ < *p <* 5 × 10^−06^. Next, we ensured independence of SNPs by clumping at a threshold of *r^2^* < 0.01. Our rationale for using a recursive *p*-value threshold was to include as many eQTLs, and thus genes, as possible in our gene prioritization strategy.

MR analyses were conducted for all identified eQTL-associated genes. Multiple testing correction was applied using Bonferroni’s method based on the number of tested genes (significance threshold α = 0.05/N_genes_). For further statistical details, refer to the *Mendelian randomization statistical methods* section of the Methods.

### Gene prioritization strategy

To prioritize the identified genes further, we selected all genes that had at least nominal eQTL MR evidence for both sociability and at least one rs-fMRI trait, as well as at least nominal CELLECT evidence for both sociability and at least one rs-fMRI trait (*see below*). We ranked these genes using the TieDIE network propagation post-propagation score percentile (*see below*). We excluded genes mapping to the major histocompatibility complex region on chromosome 6 from our prioritized list due to the complex linkage disequilibrium structure in this region and the likelihood that the genetics-based analyses will not perform well there.

#### Network propagation of genes

We used network propagation to identify additional genes affecting both sociability and the prioritized rs-fMRI traits in the context of protein–protein interaction (PPI) networks. We performed a two-seed node propagation in a tied diffusion through interacting events (TieDIE) framework to obtain genes which are close to seed genes from both sources (Paull et al., 2013). For this analysis, we selected all genes identified using FUMA that showed at least nominal eQTL MR evidence either for sociability or the rs-fMRI traits. These genes were used as seed nodes with binarized heats (i.e., if the gene was prioritized for the respective trait, the seed heat was ‘1’, otherwise it was ‘0’). As the underlying PPI network, we selected the full STRING database (Paull et al., 2013) and removed interactions in the lowest quartile of all interaction scores (confidence score < 0.309). We used the propagation algorithm implemented in the DiffuStats R package (Picart-Armada, Thompson, Buil, & Perera-Lluna, 2018) and a jack-knife (i.e., leave-one-out) procedure for each network propagation analysis to ensure robustness of results against perturbations in the seed gene list. To aid the interpretation of results from this analysis, we grouped genes into percentiles based on the mean of their post-propagation scores across each of the jack-knife permutations.

#### CELLECT cell type and gene identification

We used the CELL-type Expression-specific integration for Complex Traits (CELLECT) framework (Timshel, Thompson, & Pers, 2020) to integrate human single-nucleus RNA sequencing (snRNA-seq) datasets (Human Brain Cell Atlas v1.0, Siletti et al., 2023) with the sociability and the prioritized rs-fMRI traits GWASs. For our analysis, we selected 22 dissections from this atlas covering representative brain regions related to the DMN, based on previous evidence (Ezama et al., 2021; Smallwood et al., 2021) (see Supplementary Table S2). By leveraging partitioned LDSC and MAGMA, CELLECT identifies etiologically important GWAS-associated cell types and genes which drive the cell type-phenotype association. Our correction for multiple testing thresholds were set as follows: for CELLECT identifying cell types, we used an FDR-corrected *p <* 0.05; for CELLECT identifying genes, we used a Bonferroni threshold of *p <* 1.01 × 10^−06^ (0.05/49,689 unique genes present in the MAGMA reference data).

## Results

### Genetic overlap and causal relationship

We analyzed the genetic overlap of sociability with 31 activity (nodes) and 64 connectivity (edges) DMN-related rs-fMRI traits derived from the UK Biobank cohort (Supplementary Table S1).

#### Genetic correlations of sociability with DMN-related activity and connectivity resting-state fMRI traits

After correcting for multiple testing, none of the global genetic correlations between sociability and the 95 DMN-related rs-fMRI traits were statistically significant (Supplementary Table S3).

Even in the absence of significant genome-wide genetic correlations, local genetic correlations can be present. Therefore, we next examined the rs-fMRI traits showing nominally significant evidence for the presence of global genetic correlations (i.e., uncorrected *p* < 0.05) in more detail for local genetic correlations. Thus, we analyzed local genetic correlations between sociability and 16 DMN-related activity/connectivity rs-fMRI using LAVA. In these analyses, a strong and significant local genetic correlation was observed between sociability and spontaneous activity in the temporal cortex (node 17) at chr18:34,153,298–36,056,932 (*r_g_* = 0.66, *p* = 2.1 × 10^−4^, *p_FDR_* = 0.04) (Table 1 and Supplementary Table S4). Among the rs-fMRI connectivity traits, a strong and significant local genetic correlation was identified at chr10:114,255,955–115,588,903 (*r_g_* = −0.70, *p* = 1.7 × 10^−4^, *p_FDR_* = 0.04), linking sociability with connectivity between the frontal/cingulate and angular/temporal cortex (edge 7–11) (Table 1 and Supplementary Table S4). These areas are part of the DMN but extend to the limbic and central executive networks.

**Table 1.** Prioritized rs-fMRI traits

Rs-fMRI ID	Description	Location	Method	Effect size	*P*-value
Edge_pheno262	Pair 7–11	(Frontal|Cingulate) & (Angular|Temporal)	LAVA (local)	0.49	1.67E^−04^
Node_pheno17	Node 17	Temporal	LAVA (local)	0.44	2.11E^−04^
Edge_pheno262	Pair 7–11	(Frontal|Cingulate) & (Angular|Temporal)	MR (IVW lenient)	−0.34	9.75E^−12^
Edge_pheno1142	Pair 29–44	Parietal & Frontal_Inf	MR (IVW lenient)	0.24	1.14E^−06^
Edge_pheno816	Pair 11–36	(Angular|Temporal) & Precuneus	MR (IVW lenient)	−0.17	3.15E^−06^
Edge_pheno491	Pair 5–25	(Precuneus|Angular|Cingulate) & (Frontal|Precentral)	MR (IVW lenient)	−0.17	4.91E^−06^
Edge_pheno1698	ICA 3	Global measure	MR (IVW lenient)	−0.22	1.80E^−05^
Edge_pheno1205	Pair 5–46	(Precuneus|Angular|Cingulate) & Temporal	MR (IVW lenient)	0.18	2.18E^−05^
Edge_pheno253	Pair 7–10	(Frontal|Cingulate) & (OccipitalPrecuneus)	MR (IVW lenient)	0.16	2.92E^−05^
Node_pheno17	Node 17	Temporal	MR (IVW lenient)	0.18	1.01E^−04^^*^
Edge_pheno249	Pair 3–10	(Lingual|Fusiform) & (OccipitalPrecuneus)	MR (IVW lenient)	−0.16	1.35E^−04^
Edge_pheno593	Pair 5–29	(Precuneus|Angular|Cingulate) & Parietal	MR (IVW lenient)	−0.15	2.61E^−04^
Edge_pheno1359	Pair 21–49	Frontal_Sup & (Temporal_Mid|Angular)	MR (IVW lenient)	0.14	3.70E^−04^
Edge_pheno286	Pair 10–13	(OccipitalPrecuneus) & Frontal_Sup	MR (IVW lenient)	0.14	4.63E^−04^

*Note:* The rs-fMRI traits significantly locally genetically correlated with sociability (LAVA, see Supplementary Table S4 for further details) or with significant MR evidence for sociability (Supplementary Table S5), both after correction for multiple testing. Two traits with significant MR IVW *p*-values but evidence for pleiotropy are not shown. Effect size for LAVA: correlation *r^2^*; effect size for MR: IVW beta. The table shows uncorrected *p*-values.

* Significant MR-Egger intercept *p*-value (indicating pleiotropy). The trait is shown nevertheless, because it also has significant local genetic correlation evidence through LAVA.

#### Assessment of putatively causal effects using bidirectional MR

In the stringent bi-directional MR analysis, no results remained significant after Bonferroni correction. The putatively causal genetically proxied relationship showing the lowest nominal *p*-value (*p* = 1.42 × 10^−03^) was observed for sociability on the rs-fMRI edge 31–48 (connectivity between frontal and temporal cortex) (Supplementary Table S5, MR method ‘Inverse variance weighted’).

In the lenient bi-directional MR analysis, we found evidence for a genetically proxied causal effect of sociability on 14 rs-fMRI traits, 13 of which were connectivity measures (Table 1 and Supplementary Table S5, MR method “Inverse variance weighted correlated”). The rs-fMRI trait showing the most robust MR evidence for being causally affected by sociability was edge 7–11 (*p* = 9.75 × 10^−12^, which also showed evidence for local genetic correlation (*p* = 1.7 × 10^−4^, Supplementary Table S4). Node 17, which also exhibited significant local genetic correlation with sociability, was the only activity rs-fMRI trait significant after correction for multiple testing (*p* = 1.01 × 10^−04^). However, we found evidence for horizontal pleiotropy (intercept *p* = 6.94 × 10^−04^) in the MR-Egger analysis for this node. Because this node was independently prioritized using local genetic correlation, it was retained in the gene prioritization analyses. Two additional edges (44–49 and 10–36) that showed evidence for pleiotropy as indicated by MR-Egger (Supplementary Table S5), were excluded from further analyses, leaving 12 prioritized rs-fMRI traits.

### Gene prioritization

#### Mapping GWAS loci to genes using FUMA and MR

After establishing that sociability and 12 DMN-related rs-fMRI traits were either locally correlated (LAVA) or putatively causally related (bi-directional MR), we prioritized genes potentially affecting both phenotypes (Figure 1). Using FUMA locus-to-gene analyses for each GWAS, we mapped 83 genes for sociability and 172 unique genes across the 12 rs-fMRI traits (Supplementary Table S6). The median number of genes identified per rs-fMRI trait was 4.5, ranging from zero (edge 11–36) to 92 (edge ICA 3). Among these genes, eQTL-based MR analyses prioritized nine unique genes as significantly associated with sociability (*p* < 0.05/83) and 32 unique genes significantly associated with rs-fMRI traits (*p* < 0.05/172) (Supplementary Table S7).

#### Prioritized of cell types and genes associated with DMN and sociability

CELLECT-based cell type analyses reached the lowest *p*-values for deep-layer intra-telencephalic neurons across the different cortex dissections (Supplementary Tables S8 and S9). In addition to 54 seed genes (selected using FUMA and eQTL-MR), 139 novel genes were in the top percentile of network propagation results (Supplementary Table S10).

When combining gene prioritization methods, 43 genes showed nominal eQTL-based MR and CELLECT evidence for both sociability and at least one rs-fMRI trait (Supplementary Table S11). Most of these genes (31 of 43) had eQTLs derived in the cortex, which is likely due to the larger sample size for this tissue compared to the other brain regions. Furthermore, ten of the 43 genes were in the top decile of network propagation results, indicating they acted closely in PPI networks to the seed genes of both sociability and rs-fMRI traits (Supplementary Table S11). Only six of these 43 prioritized genes were also identified using FUMA (Table 2). Of these, only *DRD2* and *LINGO2* fulfilled all prioritization criteria, showed eQTL MR evidence significant after correction for multiple testing in any of the five tested brain regions, and were in the top percentile of network propagation.

**Table 2.** Top prioritized genes

Gene	FUMA prioritized		eQTL MR		CELLECT		TieDIE
Gene symbol	Prioritized sociability	Prioritized rs-fMRI	Min P for sociability	Min P for rs-fMRI	Min P sociability	Min P rs-fMRI	Score percentile
*DRD2*	**Yes**	No	**1.36E^−04^**	4.80E^−03^	**5.09E^−10^**	4.67E^−06^	**100**
*LINGO1*	**Yes**	No	**6.00E^−04^**	4.37E^−02^	**9.74E^−08^**	2.15E^−02^	**100**
*ELAVL2*	**Yes**	No	9.15E^−04^	3.15E^−02^	**3.15E** ^−**10**^	5.98E^−05^	**100**
*CTNND1*	**Yes**	No	7.08E^−03^	1.77E^−02^	**7.71E** ^−**08**^	2.92E^−02^	**100**
*YJEFN3*	No	No	**6.49E** ^−**05**^	2.04E^−03^	4.18E^−04^	9.65E^−03^	**98**
*AMZ1*	No	No	3.89E^−03^	2.71E^−03^	2.68E^−03^	9.56E^−04^	**98**
*LST1*	No	No	4.00E^−02^	4.32E^−03^	2.91E^−04^	1.83E^−02^	**97**
*ANKK1*	No	No	1.62E^−02^	2.54E^−02^	**3.98E** ^−**08**^	7.60E^−05^	**95**
*MIXL1*	No	No	3.69E^−03^	2.25E^−02^	1.11E^−04^	2.73E^−02^	**93**
*NOTCH4*	No	No	3.99E^−02^	2.62E^−02^	2.84E^−03^	2.12E^**−**02^	**90**
*ZIC1*	No	**Yes**	2.42E^**−**02^	3.43E^−02^	9.63E^**−**03^	3.36E^**−**06^	18
*KPNA2*	No	**Yes**	**1.78E^−04^**	**1.02E^−05^**	6.69E^**−**05^	**1.37E^−08^**	76
*MDGA1*	No	No	**2.23E^−04^**	1.93E^**−**02^	9.75E^**−**03^	1.57E^**−**03^	47
*FRAT1*	No	No	**2.64E^−04^**	1.76E^**−**02^	2.93E^**−**03^	1.21E^**−**02^	42
*SYBU*	No	No	**2.68E^−05^**	**2.29E^−04^**	2.75E^**−**06^	1.87E^**−**04^	35
*EMB*	No	No	**7.66E^−05^**	**8.28E^−05^**	1.25E^**−**03^	3.11E^**−**03^	30
*SYCE1L*	No	No	**5.29E^−04^**	2.37E^**−**02^	7.60E^**−**04^	2.88E^**−**02^	12

*Note:* From the list of 43 prioritized genes (Supplementary Table S11), the 17 genes prioritized by FUMA, showing a significant eQTL MR *p*-value after correction for multiple testing with either sociability or any of the rs-fMRI traits, exhibiting a significant CELLECT MAGMA *p*-value after correction for multiple testing, or being in the top decile of network propagation. Significant *p*-values after correction for multiple testing are shown in bold font.

Of the 43 prioritized genes, 11 were associated with anxiety and ten with depressive symptoms (Thorp et al., 2021), ten with various neuroticism items (Nagel et al., 2018), and seven with risk-taking behaviour (Thorp et al., 2021). According to the Open Targets Platform (https://www.opentargets.org), 11 of the 44 genes show an indication of being druggable, *i.e.*, fulfill any criteria for small molecule tractability.

## Discussion

In this study, we investigated the genetic and causal relationship between sociability and default mode network (DMN)-related resting-state functional magnetic resonance imaging (rs-fMRI) traits, unveiling novel insights on the neural and genetic underpinnings of social behaviour. We leveraged robust analytical frameworks including genome-wide global and local genetic correlation analyses, bi-directional Mendelian randomization (MR), and a comprehensive gene prioritization strategy. Despite the absence of significant global genetic correlations, significant local genetic correlations were found between sociability and specific DMN-related rs-fMRI activity and connectivity traits, suggesting shared pathophysiology. Subsequent analyses revealed putatively causal relationships between sociability and 12 rs-fMRI traits. Our gene prioritization approach, integrating eQTL-based MR, sn-RNAseq, and network propagation analyses, highlighted 43 putative genes with evidence supporting their influence on both sociability and DMN-related rs-fMRI traits, among which *DRD2* and *LINGO2* were most consistently implicated and emerged as key targets of interest.

Interestingly, our MR findings suggest that sociability may drive changes in DMN connectivity, which could initially appear counterintuitive. However, accumulating evidence indicates that behavioral and environmental factors can induce enduring modifications in brain structure and function (Andrews-Hanna et al., 2014; Menon, 2011). For example, early social engagement has been shown to predict long-term neural connectivity patterns. Maternal interactions during infancy are linked to functional connectivity between the DMN and salience network nearly a decade later (Degeilh et al., 2018). Similarly, increased social engagement during infancy is associated with enhanced cortical responses in regions involved in social cognition, highlighting a developmental window during which sociability shapes brain function (Jones et al., 2015). Parental internalizing and externalizing behaviors have also been shown to influence the development of children’s limbic circuits (Albar & Sattar, 2022), reinforcing the role of social environments in neural plasticity. Collectively, these findings suggest that sociability, particularly during sensitive developmental periods, can actively shape the functional organization of neural networks, including the DMN.

Our study is the first to find significant local genetic correlations between sociability and specific resting-state neural activity/connectivity traits mapping to the DMN. These traits reflect spontaneous neural activity within the superior areas of the temporal cortex (node 17) – including the superior temporal gyrus and superior temporal sulcus – and functional connectivity between the posterior/anterior cingulate cortices and the right anterior temporal lobe/right anterior angular gyrus (edge 7–11). The temporal cortex, particularly the superior temporal sulcus, is involved in the perception of social interactions. The posterior part of the superior temporal sulcus is considered one of the areas responsible for modelling the mental states, intentions, and perspectives of others (Frith & Frith, 2006). The temporal lobe’s involvement extends across various cognitive domains essential for social behaviour, including facial recognition, communication, and emotion processing (Deen, Koldewyn, Kanwisher, & Saxe, 2015). The cingulate cortex, intersecting with the DMN (through the posterior cingulate cortex) but also with the salience network (SN), is essential for detecting and orienting attention toward salient stimuli and for cognitive control modulation on a trial-by-trial basis (Bartoli et al., 2018). In particular, the anterior cingulate cortex processes social information and detects subjectively rewarding opportunities in social evaluation by assessing others’ behaviors and motivation (Apps, Rushworth, & Chang, 2016; Rigney, Koski, & Beer, 2018).

The two rs-fMRI traits showing local genetic correlations with sociability and mapping to the DMN are also functionally implicated in the auditory and language networks (node 17), central executive network, and limbic network (edge 7–11), highlighting the importance of inter-network functionality for social behaviour. Node 17 covers the entire superior temporal gyrus, which overlaps with a number of areas from both the auditory and language networks. The overlap of language and social processing is exemplified by evidence revealing a social–emotional component in semantic processing in frontal areas of the superior temporal gyrus (Mellem, Jasmin, Peng, & Martin, 2016), while the posterior superior temporal gyrus has been linked to antisocial behaviour (Raine, 2019). The central executive network is associated with high-level cognitive functions, including working memory, cognitive control, and decision-making (Steardo, D’Angelo, Monaco, Di Stefano, & Steardo, 2025). The limbic network, which is central to emotion regulation, memory, and social interactions, dynamically interacts with higher-order associative and sensory networks to support social tasks (Alcala-Lopez et al., 2018). Specific limbic regions, such as the amygdala and medial temporal structures, are structurally larger in individuals with extensive social networks, linking limbic anatomy to social engagement (Noonan, Mars, Sallet, Dunbar, & Fellows, 2018).

Only two of the 95 DMN-related rs-fMRI traits analyzed were significantly correlated with sociability in the LAVA analysis and showed significant MR evidence. However, none of the global correlation and stringent MR analyses were significant after correction for multiple testing. Given the relatively high *h^2^_SNP_* of neuroimaging traits, estimated to be between 20 and 40% for fMRI traits (Adhikari et al., 2018; Elliott et al., 2018), this low number of significant results might appear surprising. However, high *h^2^_SNP_* does not necessarily directly confer high statistical power for detecting causal loci (see Fan et al., 2018). In general, the complex and unique genetic architecture of imaging traits makes correlational analyses difficult (Toro et al., 2015). In fact, several seemingly well-powered studies have failed to detect significant genetic correlations between MRI-derived phenotypes and psychiatric traits (see Andlauer et al., 2021 and the discussion therein). Furthermore, measured rs-fMRI traits are somewhat distant from their molecular effectors because they integrate various genetic and environmental influences plus technical batches and other sources of heterogeneity (Andlauer et al., 2021. In our opinion, it is justified under these conditions to increase the search space for the causal MR analysis. It has been demonstrated that including multiple correlated instruments in an MR framework increases statistical discovery power, particularly for imaging-derived traits where few loci reach genome-wide significance (Knutson & Pan, 2021). Thus, we also adopted this approach for this study and thereby found strong evidence that genetic proxies of sociability putatively influence various DMN-related rs-fMRI measures.

Our gene prioritization strategy used triangulation – a key practice in etiological epidemiology – whereby more reliable results can be obtained when integrating analyses from different datasets and methods with orthogonal sources of bias (Lawlor, Tilling, & Davey Smith, 2016). Such an approach can be especially useful when, as is the case in this study, evidence from one method alone is not considered as robust enough. We employed triangulation to integrate diverse evidence for genes potentially affecting both sociability and DMN-related rs-fMRI measures. The validity of this approach is underscored by many highly interesting examples of genes prioritized in this study. Nevertheless, the associations of these genes should only be considered as first indications. All results presented here require replication in independent studies and using independent data.

The fact that the gene coding for the dopamine receptor D2 (*DRD2*) was highly prioritized using our approach can be considered as a positive control for our overall approach. DRD2 is the target of anti-psychotic medications and consistently appears as a significant locus across GWAS for psychiatric disorders and symptoms, including major depression (Meng et al., 2024), depressive symptoms (Nagel et al., 2018), anhedonia (Ward et al., 2019), suicide (Kimbrel et al., 2022), and addiction (Kimbrel et al., 2022). The neighboring gene, coding for the protein kinase ANKK1, was also in the top network propagation decile and is associated with, among others, major depression (Meng et al., 2024), bipolar disorder (Bipolar Disorder Working Group of the Psychiatric Genomics Consortium et al., 2023), and addiction (Hatoum et al., 2023). Notably, *ANKK1* was, unlike *DRD2*, not a network prioritization seed gene and hence identified by network propagation. Several other interesting genes already studied in the context of brain development and function, and with previous human genetics evidence, were identified in this study. One of these genes is *LINGO1*, which was, next to *DRD2*, the second gene identified across all prioritization methods. It is a regulator of large conductance Ca^2+^-activated potassium channels (Dudem et al., 2020) and is associated with depressive symptoms (Dudem et al., 2020) and addiction (Liu et al., 2019; Saunders et al., 2022). It was also reported to be associated with cognitive function in schizophrenia (Andrews et al., 2023; Fernandez-Enright et al., 2014). This gene has been suggested as a drug target (Andrews & Fernandez-Enright, 2015) but has low potential for small-molecule tractability (https://platform.opentargets.org/). Another example of an interesting prioritized gene is the regulator of protein translation *ELAVL2*, with a potential role in neurodevelopment (Mulligan & Bicknell, 2023). The *ELAVL2* gene is also associated with, among other psychiatric traits, major depression (Wainberg et al., 2022) and cognitive function (Davies et al., 2018).

To the best of our knowledge, this study was the first to find a genetic link between sociability and the DMN, expanding previous evidence of phenotypic associations (Li, Mai, & Liu, 2014; Saris et al., 2022). To this end, we leveraged the largest GWAS summary statistics available for both sociability and the rs-fMRI traits. Another strength of our study was the integration of various analysis methods, with the aim of complementing and triangulating evidence. To overcome issues inherent to imaging genetics, we used previously validated instruments to increase the statistical power of our causal inference methods. We cautiously interpret the observed MR results as supportive of a potential causal relationship while acknowledging that they are conditional on the validity of the instrumental variable assumptions (Sanderson et al., 2022). Further replication and validation in diverse samples will be necessary to strengthen confidence in these findings. From a mechanistic perspective, our findings may reflect the involvement of DMN nodes in social cognition, potentially mediated by molecular pathways implicated in dopamine signaling and synaptic plasticity. These processes could be relevant to clinical outcomes in conditions featuring social dysfunction (e.g., schizophrenia or mood disorders). However, whether targeted interventions modulating sociability would affect clinically meaningful changes in DMN connectivity remains speculative. Still, these data may guide early-stage research into potential prophylactic or therapeutic interventions.

There are also limitations to our study. The statistical power of our genetic correlation analyses was likely reduced by the complex genetic structure of both sociability and the rs-fMRI traits, as e.g. evidenced by the low *h^2^_SNP_* of the sociability GWAS (~6%). Second, the CELLECT framework has the inherent limitation to only identify genes whose increased expression affects a given trait, but not genes whose decreased expression affects either sociability or the rs-fMRI traits. The sample sizes available for eQTL analyses differed strongly between brain regions and were lower for subcortical regions compared to the cortex. Therefore, the statistical power to detect eQTLs specific to subcortical regions was lower in our analysis. While using the largest available eQTL dataset for subcortical eQTLs, we should acknowledge this as a limitation of our study. Third, our study did not aim to establish specificity of the DMN, but rather to examine its genetic associations with sociability based on an *a priori* hypothesis. The DMN was selected due to its well-documented role in social cognition and self-referential processes (Andrews-Hanna et al., 2014; Buckner et al., 2008; Ronde, van der Zee, & Kas, 2024). This hypothesis-driven approach also limited the multiple testing burden while ensuring a focused investigation of DMN-related rs-fMRI traits. Importantly, our findings do not imply that genetic influences on sociability are exclusive to the DMN. In fact, several of the identified DMN-related rs-fMRI traits, which showed genetic associations with sociability, partly overlap with other large-scale brain networks, such as the limbic, auditory, language, and central executive networks, aligning with evidence demonstrating that functional brain networks do not operate in isolation but interact dynamically (Shaw et al., 2023). However, our study was explicitly designed to test the genetic links between sociability and the DMN, and a broader examination of non-DMN networks lies beyond our predefined scope. Future studies could extend this work by investigating the broader involvement of non-DMN networks, including those partially represented by our selected traits. In terms of generalizability, our findings are derived primarily from individuals of European ancestry in the UK Biobank; therefore, their applicability to other populations or across different developmental stages remains to be determined, and replication studies in diverse populations and age groups will be essential. In the MR analyses, we could not rule out the presence of horizontal pleiotropy, i.e., the observed effect being mediated through an unrelated pathway. However, we used methods like MR-Egger regression to assess the likelihood of pleiotropy taking place and filtered the results accordingly. To this end, we employed an expansion of the standard MR-Egger method to account for weakly correlated instruments (Burgess, Dudbridge, & Thompson, 2016). Finally, the GWAS of sociability and rs-fMRI traits used in the bidirectional MR analysis were derived using participants in UK Biobank, and sample overlap is a potential concern in two-sample MR (so-called ‘winner’s curse bias’). However, previous papers on this subject have indicated that, in such a case, weak-instrument bias is amplified, and the inflation of the false discovery rate is moderate to low (Sadreev et al., 2021). To partially address and mitigate the effects of weak-instrument bias, we corrected for multiple testing using a more stringent threshold (Bonferroni’s threshold instead of FDR), and we also ensured that all instruments were subject to an F-statistic threshold. Nevertheless, we caution that this measure is likely not sufficient to alleviate any potential bias in the causal effect estimates (Burgess, Davies, & Thompson, 2016).

## Conclusions

In conclusion, our study identified genetic factors common to sociability and functional measures of DMN activity and connectivity. The significant rs-fMRI nodes and edges primarily cover the temporal, cingulate, and frontal cortex, which are brain regions integral to the DMN. However, several of these traits also overlap with other large-scale brain networks, including the limbic, auditory, language, and central executive networks, indicating that the genetic influences of sociability extend beyond the DMN. Because of the limitations of this study and the partly only nominally significant findings, all results require replication in independent studies. Nevertheless, this work conceptionally paves the way for further exploration into the clinical translation of the link between sociability and the DMN, holding the potential to inform future studies and research on novel therapeutic strategies for neuropsychiatric disorders featuring social impairment.

## Supporting information

Fanelli et al. supplementary material Fanelli et al. supplementary material

## Data Availability

All GWAS summary statistics for sociability are publicly available from Bralten et al. (2021) at https://doi.org/10.17026/dans-ztj-zga6. The rs-fMRI GWAS data from Zhao et al. (2022) can be accessed at https://zenodo.org/records/5775047. The code used for our analyses followed publicly available tutorials and documentation for the relevant software packages (e.g., the TwoSampleMR R package, LAVA, and CELLECT).

## Supplementary material

The supplementary material for this article can be found at http://doi.org/10.1017/S0033291725000832.
